# Fabrication and Characterisation of 3D-Printed Triamcinolone Acetonide-Loaded Polycaprolactone-Based Ocular Implants

**DOI:** 10.3390/pharmaceutics15010243

**Published:** 2023-01-11

**Authors:** Febri Annuryanti, Juan Domínguez-Robles, Qonita Kurnia Anjani, Muhammad Faris Adrianto, Eneko Larrañeta, Raghu Raj Singh Thakur

**Affiliations:** 1 Medical Biology Centre, School of Pharmacy, Queen’s University Belfast, 97 Lisburn Road, Belfast BT9 7BL, UK; 2 Faculty of Pharmacy, Airlangga University, Nanizar Zaman Joenoes Building, C Campus, Mulyorejo, Surabaya 60115, Indonesia

**Keywords:** triamcinolone acetonide, polycaprolactone, intravitreal, implant, 3D bioscaffold printing

## Abstract

Triamcinolone acetonide (TA) is a corticosteroid that has been used to treat posterior segment eye diseases. TA is injected intravitreally in the management of neovascular disorders; however, frequent intravitreal injections result in many potential side effects and poor patient compliance. In this work, a 3D bioprinter was used to prepare polycaprolactone (PCL) implants loaded with TA. Implants were manufactured with different shapes (filament-, rectangular-, and circle-shaped) and drug loadings (5, 10, and 20%). The characterisation results showed that TA was successfully mixed and incorporated within the PCL matrix without using solvents, and drug content reached almost 100% for all formulations. The drug release data demonstrate that the filament-shaped implants (SA/V ratio~7.3) showed the highest cumulative drug release amongst all implant shapes over 180 days, followed by rectangular- (SA/V ratio~3.7) and circle-shaped implants (SA/V ratio~2.80). Most implant drug release data best fit the Korsmeyer–Peppas model, indicating that diffusion was the prominent release mechanism. Additionally, a biocompatibility study was performed; the results showed >90% cell viability, thus proving that the TA-loaded PCL implants were safe for ocular application.

## 1. Introduction

Ocular health possesses a significant effect on people’s well-being and economic growth. The World Report on Vision reported that at least 2.2 billion people had blindness or severe vision impairment [1]. Damage to the posterior segment of the eye is typically the cause of irreversible blindness, and the treatment of this disease remains a challenge for formulation scientists due to the unique characteristics of the eye [2].

Generally, posterior eye segment diseases can be treated via topical, systemic, or periocular routes [3]. However, all these routes have some limitations, resulting in an insufficient level of drug at the target site. Topical dosage forms often fail to deliver drugs to the target site of action because of the short residence time in the cul-de-sac, and consequently demonstrate poor bioavailability of the drug, usually less than 5% [4]. Systemic drug delivery requires high doses to be administered because of the blood–aqueous and blood–retinal barriers, as well as rapid drug clearance from the eye. Moreover, high doses of steroids can increase harmful side effects [5].

Triamcinolone acetonide (TA) is a synthetic corticosteroid widely used in ophthalmology for treating severe posterior segment ocular diseases such as cystoid macular oedema, uveitis, proliferative vitreoretinopathy, and age-related macular degeneration [6,7,8,9,10]. TA is usually given as an intravitreal injection every 4–6 weeks or every 3 months, depends on the severity of the diseases.

Intravitreal injections constitute the most commonly used route for treating posterior ocular diseases since they provide higher bioavailability of therapeutics to the retina and other target tissues. Nevertheless, following intravitreal injections, drugs are typically cleared rapidly from the vitreous humour, therefore requiring frequent injections to maintain the therapeutic level of the drugs. As a result, the potential for occurrence of common side effects (e.g., cataracts, vitreous haemorrhage, endophthalmitis, and retinal detachment) increases [11]. A new approach to the drug delivery system is needed concerning the safety and efficacy of TA as a posterior segment eye disease treatment.

Sustained-release systems have previously been shown to deliver drugs to the targeted ocular tissue more efficiently, reduce administration frequency, reduce toxicity, and enhance patient compliance [12,13,14]. The design of a sustained-release drug delivery formulation treating ocular disease can vary from gels, nanoparticles, microparticulate, or implants [15,16]. In particular, ocular implants can be fabricated using biodegradable polymers and or non-biodegradable polymers [12,17]. Biodegradable implants are preferred because surgery is not required after release of the drug payload from the polymer matrix.

Polycaprolactone (PCL) is one of FDA approved biodegradable and biocompatible polymers that have been extensively studied and applied in sustained delivery systems [18,19,20,21]. Furthermore, PCL has excellent mechanical and physical characteristics, and ease of processing and shaping at low temperatures [22]. Several studies using PCL for ocular application have been carried out, including the fabrication of intracameral thin films for glaucoma treatment [23,24], episcleral thin film for vitreoretinopathy [25,26], and subretinal implants for the delivery of steroid drugs [27]. However, most of the drug delivery systems above involve using organic solvents during their fabrication processes, which might cause side effects, such as irritation, if they remain in the implant. In addition, PCL also has been used in implantable devices for sustained delivery of different drugs such as antibiotics [28], antipsychotic drugs [29,30]; antiplatelet agents, i.e., dipyridamole and acetylsalicylic acid [31,32,33]; non-steroidal anti-inflammatory drugs (NSAIDs) such as ibuprofen [34]; or even thyroid hormones such levothyroxine [34]. PCL has been shown to be well-tolerated in eyes and does not cause noticeable inflammation because it does not produce any acidic products of biodegradation [26]. The degradation mechanism of PCL degradation follows random chain scission of the ester group via hydrolysis [35,36,37], and can take up to 2–4 years, depending on the molecular weight of PCL [18,38]. The higher the molecular weight, the longer it takes for PCL to decompose naturally. Thus far, published articles about PCL implants for ocular application have utilised PCL with a molecular weight in the range of ~70 kDa–80 kDa. As a result, the degradation time of the implants is long due to the high molecular weight.

Three-dimensional (3D) printing is an additive manufacturing technology that can create 3D objects by deposition of materials in a layer-by-layer manner [39]. The capability of 3D printing technology to create intricate, unique, and personalised dosage forms and medical devices has recently increased its use in the pharmaceutical sector [19,28,40,41,42,43,44,45]. Numerous 3D printing techniques, including inkjet printing, fused deposition modelling, extrusion-based bioprinting, and stereolithography, are combined with different polymers to improve their acceptability and therapeutic efficacy. Polymers such as polyvinyl alcohol (PVA), polylactic acid (PLA), PCL, and gelatin methacryloyl (gelMA) can be used to fabricate different dosage forms and medical devices [46]. Despite its promise, however, the application of 3D printing technologies for ocular drug delivery applications has been limited thus far.

This study loaded TA into a mixture of high- and low-molecular-weight PCL polymers to enhance the mixing of both TA and the polymer matrix. For the first time, TA implants were fabricated using 3D printing techniques, negating the need to use organic solvents. Finally, the properties of TA-loaded PCL implants, the in vitro release, and the biocompatibility of the performed 3D printed TA-loaded implants were evaluated. Hence, this present work aims to fabricate a more efficient delivery system for TA that can prolong TA release with minimum side effects—a strategy that has potential to translate to the precision printing of ocular implants.

## 2. Materials and Methods

### 2.1. Materials

Triamcinolone acetonide (*M*w = 434.5, denoted as TA) was procured from Shanghai Ichemical Co. Ltd. (Shanghai, China). Polycaprolactone (PCL) CAPA^TM^ 6506 (*M*_W_ = 50 kDa, denoted as H-PCL) and CAPA^TM^ 2054 (*M*_W_ = 550 Da denoted as L-PCL), were supplied by Perstorp (Warrington, UK). Sodium dodecyl sulfate (SDS) and phosphate-buffered saline (PBS) tablets were purchased from Sigma-Aldrich (Dorset, UK). Methanol, acetonitrile, and dichloromethane were purchased from Merck (Darmstadt, Germany).

### 2.2. Fabrication of TA-Loaded PCL Implants

The TA-loaded PCL implant was designed using computer-aided design (CAD) software version 18.0. Three shapes of PCL implants (filament-like, rectangular, and circle shapes) were 3D-printed using a GeSiM 2.1 Bioscaffolder 3D Bioprinter (Radeberg, Germany). The implants were then cut into the desired shape (filament: 7 mm (L) × 0.5 mm (∅), rectangular: 2 mm (L) × 2 mm (W) × 1 mm (H), and circle: × 1 mm (H) × 5 mm (∅). The formulation compositions of TA-loaded PCL implants are shown in Table 1.

**Figure 1:**
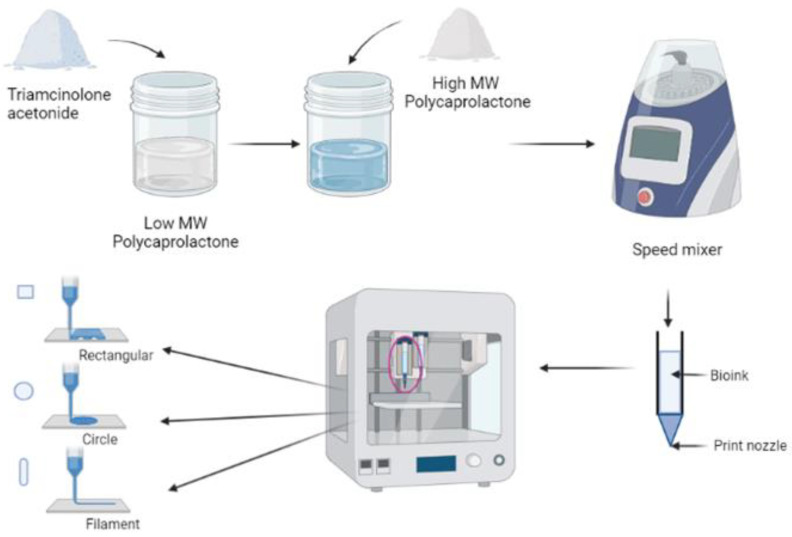
Schematic diagram of TA-loaded PCL implants fabrication using GeSiM 2.1 Bioscaffolder 3D Bioprinter.

TA was first mixed with L-PCL until a homogenous mixture was obtained; then, H-PCL was immediately added to the mixture. As L-PCL is liquid at room temperature, it can be used to help mix TA with H-PCL, resulting in a homogeneous paste. Therefore, L-PCL allows better TA integration within the thermoplastic matrix without using any solvents, as required by other manufacturing techniques such as electrospinning. The mixture was then homogenised at 5000 rpm for 5 min using a SpeedMixer^TM^ DAC 150.1 FVZ-K (Bucks, UK) and subsequently loaded into the high-temperature piston extruder of the 3D bioprinter, ready to 3D print the different implants. The feed rate was set at 10 µm/s and the extruder temperature was set at 60 °C (Figure 1).

### 2.3. Characterisation of TA-Loaded PCL Implants

Chemical interactions between the components of TA-loaded PCL implants were evaluated using Spectrum Two FTIR (Perkin Elmer, Waltham, MA) at room temperature. The IR spectra were recorded in the range of 4000–600 cm^−1^ and analysed using Spectrum 10 software. The resolution and the number of scans to record IR spectra were 4 cm^−1^ and 32, respectively.

The surface morphology of TA-loaded PCL implants was evaluated using scanning electron microscopy (SEM) (Hitachi TM3030; Tokyo, Japan). The observation condition was set to EDX mode. Implants were sectioned accordingly and mounted on HITACHI SEM Cylinder Specimen Mounts.

The thermal properties of the 3D-printed implants with and without TA were evaluated. For this purpose, thermogravimetric analysis (TGA) was performed to measure the weight loss of the 3D-printed ocular implants. TGA was performed using a Q50 Thermogravimetric analysis (TA instruments, Bellingham, WA, USA). Scans were run from 25 to 500 °C, at the heating rate of 20 °C/min under a nitrogen flow rate of 40 mL/min. Moreover, a Q20 differential scanning calorimeter (DSC) (TA instruments, Bellingham, USA) was used to establish if the TA was crystalline or amorphous within the performed 3D-printed ocular implants. Scans were run from 20 °C to 300 °C at 20 °C/min under a nitrogen flow rate of 50 mL/min.

### 2.4. Scaffold Dimensions Characterisation

The surface area and volume of the implants were calculated based on the dimension of the implants. The surface-area-to-volume (SA/V) ratio was calculated by dividing the surface area by the volume of the implants. This study was completed in triplicate (n = 3).

### 2.5. Drug Content Analysis

TA-loaded PCL implants from each formulation were randomly chosen and accurately weighed before sample preparation. Samples were dissolved in 1.0 mL of a solvent mixture (methanol:dichloromethane = 3:5) and vortexed until thoroughly dissolved. Subsequently, samples were placed in the fume hood until solvent was completely evaporated. 1 mL of methanol was added to samples, followed by centrifugation at 5000 rpm for 15 min. A hundred microliters of supernatant were taken and diluted with methanol until 1.0 mL. Finally, sample solutions were filtered through a 0.22 µm membrane filter before being injected into the HPLC system.

The HPLC system was set in an isocratic system with acetonitrile; water (45:55% *v*/*v*) used as the mobile phase. The flow rate was set at 0.9 mL/min and the temperature was maintained at 35 °C. The separation was performed using an Infinity Poroshell 120 EC-C18 column (4.6 × 250 mm, 4 µm) (Agilent, Santa Clara, CA, USA). The chromatogram was recorded at a wavelength of 236 nm and the injection volume was 20 µL. All measurements were performed in triplicate. The percentage of drug loaded was determined with Equation (1):

(1) $$\%\;\mathrm{drug}\;\mathrm{content}=\frac{\mathrm{Actual}\;\mathrm{drug}\;\mathrm{content}}{\mathrm{Theoretical}\;\mathrm{drug}\;\mathrm{amount}}\times 100\%$$

### 2.6. In Vitro Drug Release Study

An in vitro drug release study was carried out in glass vials filled with 4.0 mL of 0.5% *w*/*v* sodium dodecyl sulfate (SDS) in PBS buffer pH 7.4 containing 0.05% *w*/*v* sodium azide, in order to maintain sink conditions. The glass vials were then stored in an incubator shaker that was shaken at 40 rpm and maintained at 37 °C. All of sample solutions were collected and replaced with 4.0 mL fresh media at predetermined times. Sample solution was filtered using 0.22 µm membrane filter and to be analysed using HPLC system as used in TA content determination. The cumulative percentage release of TA was plotted against time. Experiments were conducted in triplicate (n = 3).

The release kinetics of TA from each formulation was analysed using the Higuchi (Equation (2)) and Korsmeyer–Peppas (Equation (3)) mathematical models. Only the portion of the graph showing up to 60% drug release was fitted to the Korsmeyer–Peppas model.

(2) $$\frac{Q_{t}}{Q_{\infty }}=K_{H}t^{0.5}$$

(3) $$\frac{Q_{t}}{Q_{\infty }}=K_{KP}t^{n}$$

where *Q_t_/Q_∞_* is the fraction of drug released at time t, K_H_ is the Higuchi dissolution constant, and K_KP_ is the Korsmeyer–Peppas constant. The value of the exponent “n” in the Korsmeyer–Peppas model can be used as an indication of the release mechanism [47,48]. If the obtained “n” value is around 0.5, this indicates that the drug is been released following Fickian diffusion [47,48]. On the other hand, if the obtained “n” value ranges between 0.5 and 1, it indicates anomalous (non-Fickian) transport [49,50,51]. Finally, when the “n” value is around 1, it indicates case II transport [47,48].

### 2.7. Implants Degradation

Degradation of PCL implants was evaluated by weighing the initial dry implants (W_o_), immersing them in the release media, incubating at 37 °C, and shaking at 40 rpm. At the end of the degradation study, the implants were dried overnight under vacuum conditions at room temperature. Dried implants were then weighed and recorded as W_t_. The percentage weight remaining was calculated using Equation (4):

(4) $$\mathrm{mass}\;\mathrm{remaining}=\frac{\mathrm{Initial}\;\mathrm{weight}\;\left(\mathrm{Wo}\right)}{\mathrm{Final}\;\mathrm{dry}\;\mathrm{weight}\;\left(\mathrm{Wt}\right)}\times 100\%$$

SEM analysis was performed to observe the surface morphology of the implants at the end of the release study.

### 2.8. In Vitro Biocompatibility Study

#### 2.8.1. Cell Culture

Human retinal pigmen epithelial (ARPE-19) cells were used for the biocompatibility study. ARPE-19 cells were cultured in an optimised medium comprised of Dulbecco’s modified Eagle’s Medium DMEM/F-12 (Dulbecco’s Modified Eagle Medium/Nutrient Mixture F-12), modulated with HEPES/glutamine and enriched with 10% heat-inactivated foetal bovine serum and 1% penicillin/streptomycin stock solution (10,000 units/mL penicillin and 10 mg/mL streptomycin). Cells were then incubated in humified conditions (5% CO_2_/air) at 37 °C.

#### 2.8.2. Cell Passage

ARPE-19 cells were cultured in T75 cm^2^ media consisting of DMEM/F-12, modulated with HEPES/glutamine and enriched with 10% heat-inactivated foetal bovine serum and 1% penicillin/streptomycin stock solution (10,000 units/mL penicillin and 10 mg/mL streptomycin); complete media change was performed every 2–3 days. At the third passage, the cell pellet was seeded in a 96-well tissue culture plate with a seeding density of 2 × 10^4^ cells/well. Finally, cells were incubated for 36 h before the biocompatibility study.

#### 2.8.3. Sample Preparation

In this study, filament-shaped implants were used to evaluate the in vitro biocompatibility of the samples. The test was carried out using indirect and direct assay methods according to ISO 10993-5:2009—Biological evaluation of medical devices. For the indirect assay, implants were dipped in 70% ethanol for sterilisation purposes, immersed in 4 mL of DMEM/F12 containing 1.2% streptomycin/penicillin, and stored for 24 h in an incubator. Finally, 200 µL of sample solution was pipetted into pre-incubated TCPs and incubated for 24 h. For the direct assay, implants were placed on top of ARPE-19 cells in 96-well plates. Subsequently, 200 µL of fresh media was added and incubated for 24 h at 37 °C. After incubation, the 96-well plate was inverted to remove the implants.

#### 2.8.4. Cell Viability Assay

Cell viability assay was performed based on resazurin reduction assay. Twenty microliters of resazurin sodium salt solution was added to 200 µL samples in a 96-well plate photometer and incubated for 4 h. Samples were then analysed using fluorescence at 545 nm, and cell viability was calculated based on the optical density (OD) by using Equation (5). DMEM/F12 and DMSO were used as negative and positive controls, respectively. All experiments were performed in triplicate (n = 3).

(5) $$\mathrm{Cell}\;\mathrm{viability}\left(\%\right)=\frac{\mathrm{ODsamples}\;}{\mathrm{ODnegative}\;\mathrm{control}}\times 100$$

### 2.9. Statistical Analysis

Data were analysed with GraphPad Prism^®^ version 9 (GraphPad Software, San Diego, CA, USA) and Microsoft Excel. The results are presented as means ± standard deviation (SD). The comparative study was performed using a t-test and one-way variance analysis (ANOVA). Significant difference was accepted with a *p*-value < 0.05.

## 3. Results and Discussion

### 3.1. Fabrication and Characterisation of TA-Loaded PCL Implants

**Figure 2:**
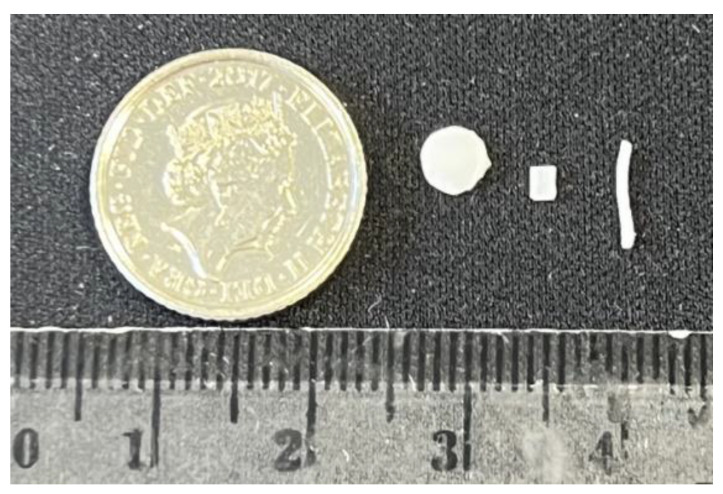
Different shapes of TA-loaded PCL implants printed using GeSiM 2.1 Bioscaffolder 3D bioprinter.

PCL is one of the FDA-approved biodegradable polymers for developing drug delivery systems due to its biocompatibility and biodegradability [18,19,20,21]. TA-loaded PCL-based implants were successfully manufactured using semi-solid extrusion (SSE) 3D printing technology. Implants were printed in filament-like, rectangular, and circular shapes (Figure 2) using all the formulation compositions listed in Table 1.

**Figure 3:**
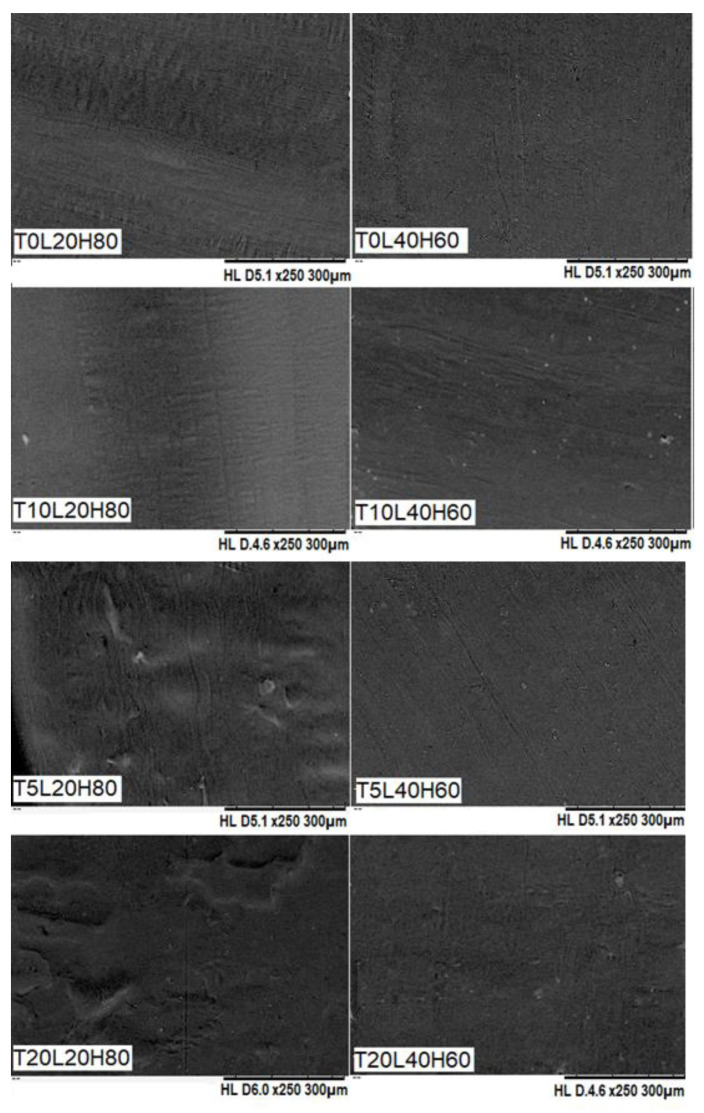
SEM images of blank PCL implants and TA-loaded PCL implants.

SEM was used to confirm the surface morphology of TA-loaded PCL implants. As shown in Figure 3, the surface of PCL-based implants was smooth and homogenous, and no visible drug crystals or aggregates were found on their surfaces, indicating that TA is miscible and evenly distributed throughout the PCL matrix. Additionally, the surface of the blank PCL implants (T0L20H80 and T0L40H60) was also smooth and homogeneous, thus showing that both H-PCL and L-PCL were evenly mixed. Overall, these results indicate the capability of the SSE 3D printing technology to produce homogenous solid implants with a vast range of doses.

**Figure 4:**
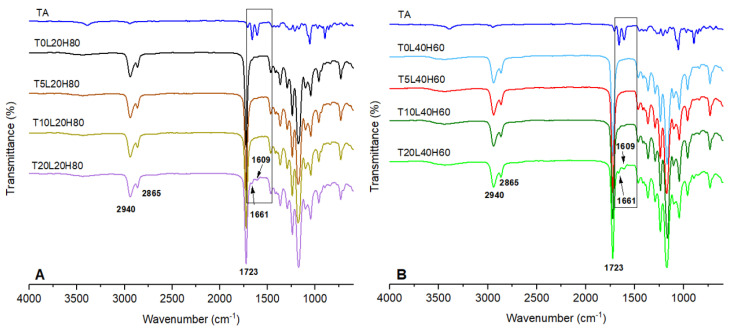
FTIR-spectra of (**A**) pure TA, blank PCL (T0L20H80), and TA-loaded in a matrix containing 20% L-PCL and 80% H-PCL, (**B**) pure TA, blank PCL (T0L40H60), and TA-loaded in a matrix containing 40% L-PCL and 60% H-PCL.

ATR-FTIR analysis was investigated to define the chemical interaction between the PCL and TA. The ATR-FTIR infrared spectra of TA and blank PCL matrix implants were compared with the TA-loaded PCL implants, as presented in Figure 4. TA exhibited a characteristic infrared absorption band at 3395 cm^−1^ and 2948 cm^−1^ that linked to the stretching vibration of the OH and C-H groups, respectively. Moreover, the absorption band at 1707 cm^−1^ represents the C=O stretching vibration of this drug. The band located at 1661 cm^−1^ and 1609 cm^−1^ corresponds to the stretching vibration of C=C bonds. Other specific infrared peaks of TA were located at 1171 cm^−1^ and 1056 cm^−1^, and were attributed to the C-O-C bond in aliphatic esters and the stretching vibration of C-F, respectively. The bands of TA found in this study were comparable to those previously obtained in other works [6,26,52]. Blank PCL matrices are composed of two PCL polymers with varying molecular weights. Moreover, both blank PCL-based implants showed a strong carbonyl stretching vibration at 1723 cm^−1^.

Moreover, the bands located at 2940 and 2865 cm^−1^ represent the stretching vibration of C-H bonds. The band located at 1180 cm^−1^ represents C=O=C stretching. Another characteristic PCL band can be found at 1293 cm^−1^, associated with C=O and C-C stretching in the crystalline phase of PCL [38,53,54,55]. All spectral characteristics of PCL were observed in TA-loaded implants, although TA peaks were not observed when the drug loading was low. This phenomenon has also been documented in several publications [56,57]. When the drug loading is low, the bulk polymer is anticipated to conceal or obscure the FTIR contributions of the drug at low loading levels [58]. However, by increasing the drug loading, peaks at 1661 cm^−1^ and 1609 cm^−1^ were identified, in addition to those of PCL. These characteristics could be used to determine the presence of TA in implants. Moreover, no new peaks were observed, suggesting no chemical reactions occurred during the 3D-printing process.

**Figure 5:**
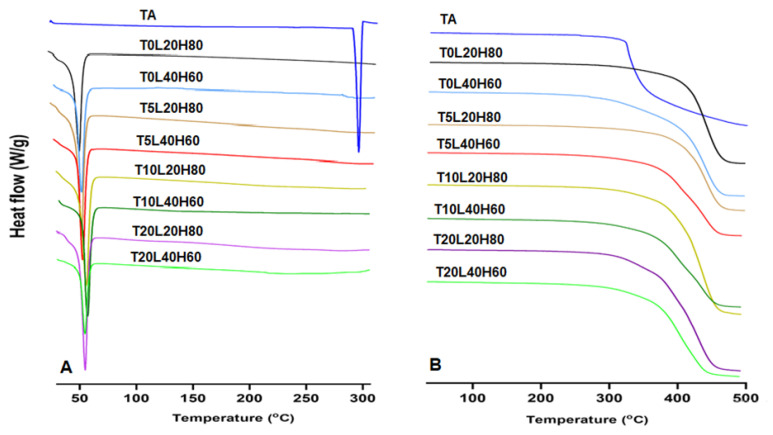
(**A**) DSC profile and (**B**) TGA data of pure TA, blank PCLs, and TA-loaded PCL implants.

DSC analysis of the pure TA, blank PCL implants, and TA-loaded PCL implants was performed to understand TA incorporation into the polymer (Figure 5A). Both blank PCL implants (T0L20H80 and T0L40H60) showed an endothermic peak between 54 to 59 °C [18,34,55]. Moreover, a sharp endothermic peak at 290 °C corresponds to the melting temperature of TA, as previously reported [21]. This endothermic melting peak of TA was not observed in any of the TA-loaded PCL implants, thus indicating that the crystalline form of TA was converted to the amorphous form after being combined with the PCL matrix (H-PCL and L-PCL). Similar outcomes have already been reported for different drugs such as dipyridamole (DIP) [31] or acetylsalicylic acid (ASA) [33], as well as for other types of polymeric matrix such as thermoplastic polyurethane (TPU) [32].

TGA data (Figure 5B) were obtained to assess the thermal stability and degradation profiles of pure TA, blank PCL implants, and TA-loaded PCL implants. TGA results showed that TA started degrading at temperatures slightly above 300 °C. Moreover, as expected, the PCL blank implant containing the highest percentage of L-PCL (T0L40H60) started degrading at temperatures around 300 °C. In contrast, the PCL blank implant containing the highest percentage of H-PCL (T0L20H80) showed a higher T_onset_ (around 400 °C). Therefore, the T_onset_ of the TA-loaded PCL implants was shifted lower when a higher percentage of L-PCL and/or TA was added.

### 3.2. TA Content Analysis

**Figure 6:**
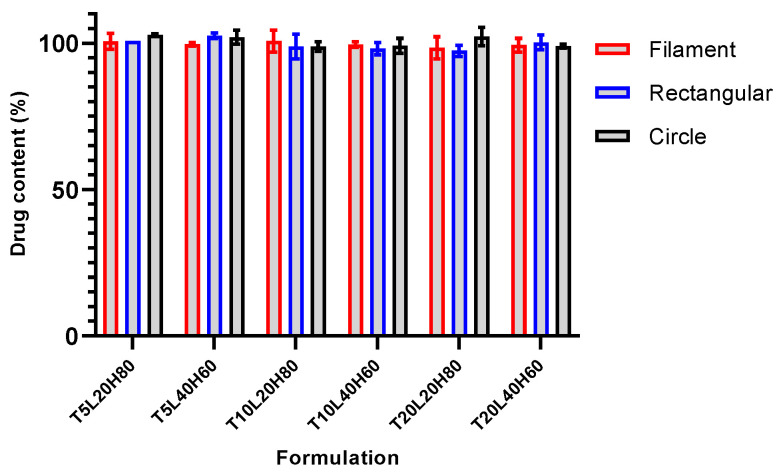
Results of drug content analysis of TA-loaded PCL implants. Data presented as mean ± SD, n = 3.

Ensuring that the chosen fabrication method of TA-loaded PCL implants is capable of producing samples with uniform drug distribution throughout the sample is critical to ensure controlled drug release [6]. Implants with different shapes from each formulation were randomly selected and analysed using the optimised HPLC method. The mean value of relative drug content from tested implants was 99.98 ± 1.60%, with a *p*-value > 0.5236. The results of the uniformity content confirmed that the TA was distributed uniformly throughout the polymer matrix (Figure 6). The results indicate that the 3D printing implant manufacturing technique is reproducible and produces a uniform drug distribution in the PCL matrix.

### 3.3. In Vitro Release Study

**Figure 7:**
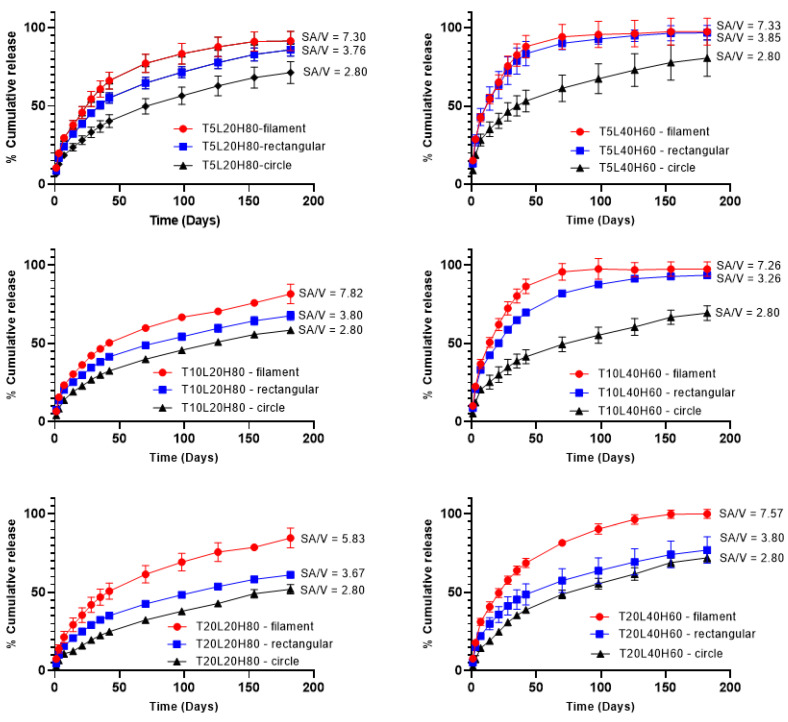
In vitro cumulative release of TA from PCL implants over 180 days. Results represent means ± SD (n = 3).

In vitro release studies were conducted using PBS buffer (pH 7.4) containing 0.05% *w*/*v* sodium azide and 0.5% *w*/*v* SDS. Sodium azide was added to the media to prevent microbial growth during the in vitro release studies [59,60,61,62]. In addition, SDS can increase TA solubility by up to 14 times [63]. Figure 7 shows the cumulative drug release of all formulations over 180 days. As expected, formulations with a higher ratio of L-PCL (L40H60) released TA faster than those with a higher H-PCL ratio (L20H80). Therefore, implants containing a higher ratio of L-PCL exhibited two different release stages, and this effect was much less pronounced (or even non-existent) when implants contained a higher ratio of H-PCL. H-PCL has a longer chain length than L-PCL; thus, it is more hydrophobic than L-PCL. Additionally, a higher ratio of L-PCL in formulations may provide better interactions with release media so that TA can be quickly released from the implants.

The surface-area-to-volume ratio effect on drug release was also evaluated using different implant shapes (Table 2). The filament implant has the highest surface-area-to-volume ratio (SA/V~7.30), followed by the rectangular shape (SA/V~3.70), and, finally, the circle shape (SA/V~2.80). The dissolution tests show that the highest SA/V ratio corresponds to the highest cumulative release, which is crucial in defining drug release profiles. These results follow the studies by Goyanes et al. and Reynolds et al. [64,65]. Furthermore, statistical analysis was performed to see any significant effect of the SA/V ratio on the cumulative drug release. A one-way ANOVA study showed significant differences in cumulative release amongst implant shapes in each formulation (*p* < 0.0001).

The percentage cumulative release data were fitted to the Higuchi and Korsmeyer–Peppas models to assess drug release mechanism (Table 3). Most samples show high regression coefficient (R^2^) values for Korsmeyer–Peppas, with different n-values. Rectangular-shaped implants showed an n-value less than 0.5, indicating that the release mechanism is based on Fickian diffusion. In the Fickian diffusion mechanism, the solvent diffusion rate determines the drug release, rather than the polymer relaxation rate. These results agreed with previous reports [51,66,67]. On the other hand, the filament- and circle-shaped showed n-value lower than 0.5, suggesting a pseudo-Fickian diffusion mechanism [68]. Interestingly, in some cases, the n-values were slightly higher than 0.5, indicating an anomalous transport mechanism, which is the release is not only governed by diffusion but also influencing by the relaxation of polymeric chains [49,50,51]. This behaviour was found only in some L40H60 samples. This suggests that the presence of a higher content of low molecular weight PCL can contribute to matrix relaxation. Despite these small differences between the implants, all the n-values obtained were close to 0.5, thus suggesting that TA relies heavily on Fickian diffusion to be released from the implants. Considering that implants were made of PCL, which degrades slower, it is not surprising that the drug is released predominantly by diffusion, as has been reported in the past for PCL-based implants [29,69].

### 3.4. Implant Degradation

Implant degradation, quantified by percentage mass loss, was also investigated during in vitro release. Table 4 depicts the mass loss of all implant formulations over 180-days, with a higher mass loss observed from implants containing a higher ratio of L-PCL (L40H60). Further statistical analysis was performed to observe the parameters (drug loading or polymer ratio) that governed the mass loss of PCL implants. The statistical analysis results showed that different polymer ratios affect TA release from the implants, with the *p*-value (0.0027) < 0.5 for all drug loadings; whereas different drug loadings have no significant effect on mass loss (*p* value = 0.1357).

**Figure 8:**
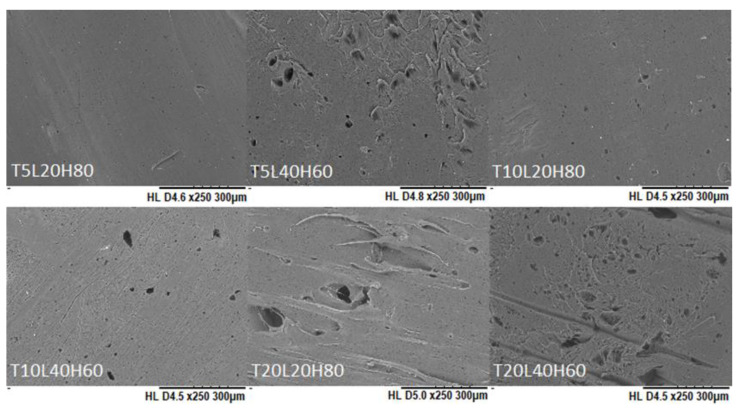
SEM images of PCL implants after in vitro release over 180 days.

The degradation rate of PCL not only depends on the morphological and structural formation, but is also influenced by the surface-area-to-volume ratio [70,71]. A higher SA/V ratio improves the penetration of release media into the implants and initiates the chain scission rate [72]. Therefore, a higher mass loss was observed from filament-shaped implants, which have the highest SA/V ratio. The changes in the surface morphology of PCL implants after in vitro release was also observed using SEM (Figure 8). Unlike TA-loaded PCL implants on day 0 (Figure 3), TA-loaded PCL implants showed a more porous structure after 180 days. Accordingly, we investigated pore size measurements for all implants after the drug release study (Table S1, Supplementary Data).

### 3.5. Biocompatibility Study

**Figure 9:**
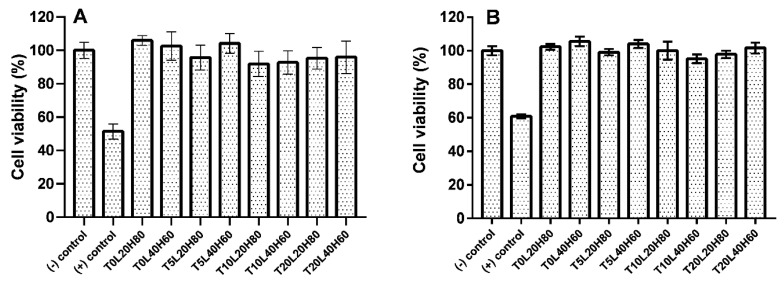
The cell viability of ARPE-19 cells by resazurin reduction assay after being treated with TA-loaded PCL implants: (**A**) indirect assay method and (**B**) direct assay method. Results represent means ± SD (n = 3).

Filament-shaped implants were used for the biocompatibility study because this shape is more feasible for further application. An indirect biocompatibility assay was completed by calculating the viability of ARPE-19 cells after exposure to the release media. In contrast, the direct assay involved the placement of the implant on top of the ARPE-19 cells. According to ISO standards, a cytotoxic effect is considered when the percentage of cell viability is below 70% [73]. The results of the cell viability study are shown in Figure 9. The resazurin assay showed that the cells had more than 90% viability, indicating that implants were non-cytotoxic. Statistical analysis showed no significant differences between the negative control and all formulations for both indirect assay (*p* value = 0.1940) and direct assay (*p* value = 0.1901). Moreover, no significant differences were found when all formulations were compared with each other (*p* value > 0.05). According to the biocompatibility result, TA-loaded PCL implants are safe for ocular application. However, further in vivo studies are required to ensure the safety of the implants.

## 4. Conclusions

This study revealed that TA-loaded PCL implants were successfully manufactured SSE using 3D printing technology. TA was selected as a drug candidate because it is an effective and safe treatment for posterior segment diseases. TA was properly incorporated within the PCL matrix (H-PCL/L-PCL), which was confirmed by SEM. The fabrication technique of TA-loaded PCL implants ensured that the drug was distributed uniformly throughout the system. The FTIR data revealed no chemical reaction between TA and PCL, which is also supported by the thermal analysis results of TGA and DSC. The development of biodegradable ocular implants capable of providing a sustained release of a corticosteroid drug, such as TA, for at least 6 months, marks a major step forward in the fight against posterior segment ocular diseases such as diabetic retinopathy. In addition, the biocompatibility study showed that the TA-loaded PCL implants were safe for ocular application, since all the formulations exhibited percentages of cell viability above 90%. Thus, TA in combination with H-PCL and L-PCL did not compromise the cell viability of ARPE-19 cells. These TA-loaded PCL implants are a valuable alternative to frequent intravitreal injections for treating posterior segment eye disease (PSED). These results thus suggest that SSE 3D printing technology can be successfully used to manufacture the abovementioned ocular implants and has great potential to be transferred to clinical applications. Furthermore, this work has shown that 3D printing technology can be used to precisely produce implants with modifications in shape and dose, allowing ocular implants to be personalised to the individual needs of each patient.

## Figures and Tables

**Table 1:** Formulation composition of TA-loaded PCL implants.

Formulation	Composition (% *w*/*w*)			L-PCL/H-PCL Ratio
	L-PCL	H-PCL	TA	
T0L20H80	20	80	0	20/80
T0L40H60	40	60	0	40/60
T5L20H80	19	76	5	20/80
T5L40H60	38	57	5	40/60
T10L20H80	18	72	10	20/80
T10L40H60	36	54	10	40/60
T20L20H80	16	64	20	20/80
T20L40H60	32	48	20	40/60

**Table 2:** Surface area, volume, and SA/V ratio of PCL implants.

Formulation	Shape	Surface Area	Volume	SA/V Ratio
T5L20H80	Filament	12.98 ± 0.27	1.78 ± 0.07	7.30 ± 0.14
	Rectangular	19.49 ± 0.66	5.18 ± 0.23	3.76 ± 0.04
	Circle	54.73 ± 1.52	19.53 ± 0.63	2.80 ± 0.01
T5L40H60	Filament	13.00 ± 0.22	1.77 ± 0.05	7.33 ± 0.07
	Rectangular	18.17 ± 0.62	4.72 ± 0.23	3.85 ± 0.05
	Circle	55.48 ± 0.58	19.84 ± 0.24	2.80 ± 0.01
T10L20H80	Filament	12.10 ± 0.60	1.55 ± 0.15	7.82 ± 0.37
	Rectangular	18.89 ± 1.91	4.98 ± 0.65	3.80 ± 0.13
	Circle	54.73 ± 1.09	19.53 ± 0.45	2.80 ± 0.01
T10L40H60	Filament	13.18 ± 0.18	1.82 ± 0.06	7.26 ± 0.12
	Rectangular	19.70 ± 0.11	5.23 ± 0.02	3.76 ± 0.01
	Circle	54.48 ± 0.95	19.43 ± 0.45	2.80 ± 0.01
T20L20H80	Filament	13.99 ± 0.12	2.40 ± 0.04	5.83 ± 0.06
	Rectangular	21.16 ± 0.34	5.77 ± 0.12	3.67 ± 0.02
	Circle	53.48 ± 0.75	19.01 ± 0.31	2.80 ± 0.01
T20L40H60	Filament	12.56 ± 0.32	1.66 ± 0.08	7.57 ± 0.19
	Rectangular	18.78 ± 0.75	4.94 ± 0.26	3.80 ± 0.05
	Circle	54.60 ± 0.38	19.48 ± 0.16	2.80 ± 0.00

**Table 3:** Data obtained after fitting TA release curves to Korsmeyer–Peppas and Higuchi models.

Formulation	Filament			Rectangular			Circle		
	Korsmeyer–Peppas		Higuchi	Korsmeyer– Peppas		Higuchi	Korsmeyer– Peppas		Higuchi
	R^2^	n	R^2^	R^2^	n	R^2^	R^2^	n	R^2^
T5L20H80	0.9954	0.461	0.9939	0.9942	0.472	0.9851	0.9982	0.433	0.9850
T5L40H60	0.9922	0.446	0.9812	0.9796	0.475	0.9750	0.9869	0.378	0.9325
T10L20H80	0.9911	0.437	0.9679	0.9962	0.386	0.9448	0.9964	0.431	0.9834
T10L40H60	0.9925	0.552	0.9922	0.9920	0.477	0.9904	0.9863	0.411	0.9515
T20L20H80	0.9963	0.497	0.9942	0.9966	0.426	0.9771	0.9917	0.454	0.9870
T20L40H60	0.9923	0.518	0.9907	0.9856	0.437	0.9606	0.9891	0.509	0.9902

**Table 4:** Mass loss of PCL implants over 180-day release.

Formulation	Mass Loss of PCL (%)		
	Filament	Rectangular	Circle
T5L20H80	12.34 ± 0.23	11.00 ± 0.19	9.92 ± 0.62
T5L40H60	14.20 ± 0.67	14.08 ± 1.74	13.42 ± 1.32
T10L20H80	13.02 ± 0.32	12.05 ± 1.04	10.18 ± 0.36
T10L40H60	14.81 ± 1.37	14.40 ± 1.04	14.25 ± 0.20
T20L20H80	14.41 ± 0.84	13.77 ± 0.93	10.38 ± 0.70
T20L40H60	18.27 ± 2.52	16.97 ± 1.04	16.63 ± 2.00

## Data Availability

Not applicable.

## Supplementary Materials

The following supporting information can be downloaded at: https://www.mdpi.com/article/10.3390/pharmaceutics15010243/s1, Table S1: Pore size measurement of TA-loaded PCL implants after drug release study.
