# Supplementary material for: Fabrication and Characterisation of 3D-Printed Triamcinolone Acetonide-Loaded Polycaprolactone-Based Ocular Implants
Source: Pharmaceutics. 2023 Jan 11;15(1):243. doi: 10.3390/pharmaceutics15010243 (PMC9863928; doi:10.3390/pharmaceutics15010243)
Supplement: Supplementary file 1 [file pharmaceutics-15-00243-s001.zip › pharmaceutics-1996449-supplementary.pdf]

Supplementary data

Table S1. Pore size measurement of TA-loaded PCL implants after drug release study

Formulation	Pore size ± SD (μm); n = 15
T5L20H80	1.74 ± 1.25
T10L20H80	2.03 ± 1.40
T20L20H80	3.53 ± 1.70
T5L40H60	3.09 ± 0.95
T10L40H60	3.39 ± 1.18
T20L40H60	3.88 ± 1.64
